# Supplementary material for: Automatic uterus segmentation in transvaginal ultrasound using U-Net and nnU-Net
Source: PLoS One. 2025 Nov 12;20(11):e0336237. doi: 10.1371/journal.pone.0336237 (PMC12611126; doi:10.1371/journal.pone.0336237)
Supplement: Attachment — Submitted filename: response_to_reviewers.docx [file pone.0336237.s002.docx]

We would like to thank you for your constructive feedback. We have updated the paper accordingly and think the paper is much stronger as a result.

# Journal requirements

1. Style requirements

After carefully reading the style requirements manual again, we have made appropriate changes.

2. Code sharing

We have included our GitHub repository and made mention of it in line 69-70 and 416.

3. Clarification on the part of the manuscript that has been published somewhere else

Our co-author of the paper, Lisa Trommelen, published a peer-reviewed paper called Grading Sonographic Severity of Adenomyosis

(<https://onlinelibrary.wiley.com/doi/full/10.1002/jum.16612>), where she aimed to develop a semi-quantifiable sonographic method to grade the severity of adenomyosis and assess the feasibility and interobserver reliability of this method. For this, she collected data, namely two-dimensional (2D) grayscale transvaginal ultrasound video clips and 3-dimensional (3D) volumes of the uterus of 35 women. This is the data that we then used in our study for another purpose: uterus segmentation. There is no risk of dual publication, as Lisa’s study focused on obtaining these data and analyzing them in a clinical setting, whereas our study focusses on creating automatic semantic segmentations of these images without a direct clinical conclusion or implementation. This is now explained in a clearer way in the subsection ‘dataset collection’.

4. Addressing the dataset availability

Because we are using a retrospective approach with data collected by a colleague for another project, we do not have ethical approval to make this data accessible online. We do appreciate this consideration and certainly we’ll implement it in our prospective work.

# Reviewer comments

## Reviewer 1

1. Reconsidering the title

We have now reconsidered the title to be: Automatic Uterus Segmentation in Transvaginal Ultrasound using U-Net and nnU-Net.

2. Distinguishing U-Net and nnU-Net more clearly

We aimed to keep the description accessible by not delving into the technical distinctions between U-Net and nnU-Net. However, we understand the importance of accuracy in terminology and have now changed it. In the introduction (line 86-87) we have now added a sentence for clarification.

3. Include more studies on deep learning-based segmentation in ultrasound beyond obstetrics & revision about what is known about the study subject (reviewer 2)

We appreciate this comment, and we have since incorporated the latest literature reviews in this field in the introduction, as well as studies from other fields. This answer also incorporates the feedback given by reviewer 2 in comment 3.

4. Segmentation masks

We have now clarified more on the segmentation masks in the dataset collection section.

Reviewer 1, comment 5: model weights

We have now added the u-net model weights in the public GitHub repo under the file ‘unetmodels.py’ along with instructions how to use them. The nnU-Net model weights were not saved during training.

## Reviewer 2

1. Spelling and grammar mistakes

We have now fixed the additional spelling and grammar mistakes.

2. The importance of the problem and why this study was necessary

We have now changed the introduction to focus more on the importance of the problem and why our study was necessary, namely: automatic semantic segmentation has been proven to be very successful in medical imaging but in the field of gynecology, it is less advanced, especially when it comes to models trained on TVUS data. This is what our study aims to address.

3. Clarity on the study design

In the field of computer science and AI, there is no specific name for our study design. We take the following steps: we prepare our dataset, using the data collected by our co-author Lisa; we choose our model architecture; we train our model; and finally, we evaluate our model. We have now changed the materials and methods section to be clearer about the fact that we are not collecting data from patients as part of this study but merely using an already existing dataset to train our models.

4. The setting in which the study was collected

We have now added the sentence ‘The data was collected in the gynecological outpatient clinic of the Amsterdam University Medical Center.’ On line 129-130.

5. Patient characteristics and selection bias

As we were not designing the data collection, we put limited patient characteristics in this paper. However, some can be found in table 2. We have now also added the sentence ‘The study included consecutive premenopausal women with sonographic signs of adenomyosis.’ To clarify the inclusion criteria. After close inspection we didn’t identify any bias in our dataset, before moving on with the automatic analysis. We also didn’t notice any unusual results after the automatic segmentation.

6. A brief qualitative resume of the results

We have now changed our more quantitative summary to a qualitative one in the discussion.

7. Comparison to similar studies already present in literature

There are very few papers that are looking at different modalities of ultrasound scans, however, we have now included a sentence comparing our work to the recent literature reviews we have included in reviewer 1 comment 3.

8. Analyzing strengths and limitations of the study

We have now included a sentence that discusses the fact that our model was trained on a specific population of women in our limitations section, line 459-460.
